# Prevalence and Risk Factors of Genital Human Papillomavirus Infections among Women in Lagos, Nigeria

**DOI:** 10.3390/tropicalmed7110386

**Published:** 2022-11-18

**Authors:** Oluwaseyi Sedowhe Ashaka, Adesuyi Ayodeji Omoare, Ayorinde Babatunde James, Oluwapelumi Olufemi Adeyemi, Femi Oladiji, Kayode Adebamiji Adeniji, Kehinde Sharafadeen Okunade, Olajide Olubunmi Agbede

**Affiliations:** 1 Department of Biological Sciences and Biotechnology, Caleb University, Lagos 106102, Nigeria; 2 Department of Medical Microbiology and Parasitology, College of Health Sciences, University of Ilorin, Ilorin 240003, Nigeria; 3 National Reference Laboratory Department of Public Health Laboratory Services (PHLS), Nigeria Centre for Disease Control and Prevention (NCDC), Gaduwa, Abuja 900110, Nigeria; 4 Department of Biochemistry & Nutrition, Nigerian Institute of Medical Research, Lagos 100001, Nigeria; 5 Department of Epidemiology and Community Health, University of Ilorin, Ilorin 240003, Nigeria; 6 Department of Morbid Anatomy and Histopathology, College of Health Sciences, University of Ilorin, Ilorin 240003, Nigeria; 7 Lagos University Teaching Hospital, Lagos 101014, Nigeria

**Keywords:** human papillomavirus, prevalence, risk factors, cytology, Lagos

## Abstract

Regional variations exist in HPV prevalence worldwide despite reports of high prevalence rates among African women. Limited data on genital HPV prevalence necessitated this study with the aim of determining the prevalence of genital HPV and associated risk factors among women in Lagos, Nigeria. Exfoliated cervical cells were collected with consent from 165 women using a cervical brush. Viral DNA was extracted and amplified by nested PCR using two sets of consensus primers (MY09/11 and GP5+/6+). An unconditional logistic regression model was used to identify predictors of HPV positivity. The HPV prevalence was 81.82% in all women and 87.59% in women with normal cytology. The risk of HPV infection was significantly increased among women who had a history of STI (odds ratio (OR) 3.94; 95% confidence interval (CI): 1.51–10.25, *p* = 0.005) while there was a significantly reduced risk of HPV infection among those who used condoms (odds ratio (OR) 3.94; 95% confidence interval (CI): 0.18–0.91, *p* = 0.03). The HPV prevalence observed shows an increased transmission of the virus in Lagos, Nigeria. Therefore, there is a need for intense public awareness and the implementation of early detection tests, treatment, and vaccination to prevent an increase in cervical cancer cases in Lagos, Nigeria.

## 1. Introduction

Genital human papillomavirus (HPV) infections with a global prevalence of about 11–12% have been ranked as one of the most common viral agents of sexually transmitted infections worldwide [1]. Human papillomaviruses are double-stranded DNA viruses devoid of an envelope. These viruses infect mucosal and cutaneous epithelia. Over 100 genotypes of HPV have been identified, of which a number infect the genital tract. There has been evidence linking HPV to the development of epithelial lesions which may be benign or malignant [2].

The estimated prevalence of HPV in different regions of the world differs. Compared to other regions of the world, the highest prevalence of 22.1% is reported in Africa [3]. There are also variations in the prevalence rates reported in different African countries depending on the population being considered. In Sudan and Senegal, HPV prevalence has been reported to be 2.2 and 18%, respectively [4,5]. Moreover, among women in South Africa, Burkina Faso, Zimbabwe, and the Benin Republic a prevalence of 20–33% has been reported [6,7,8,9]. Higher prevalence figures of 40% and above have been reported in places such as rural Mozambique, Nairobi, Kenya, Morocco, and Zambia [10,11,12,13].

In Nigeria, HPV prevalence has been reported to vary in different parts of the country. It ranges from 10.0% in Irun, Ondo State to 21.6% in Ile-Ife, Osun State and 26.3% in Ibadan, Oyo State [14,15,16]. Prevalence rates of 30.4 and 36.5% were reported by Adegbesan-Omilabu et al. [17] and Okunade et al. [18], respectively. It has been observed that the method used in most studies contributed to the prevalence obtained [19].

Some risk factors that have been linked to the increased prevalence of HPV infection include early onset of sexual activity, sexual promiscuity, an increasing number of lifetime sexual partners, and poor hygienic conditions [18,20]. Research has shown that HPV disease can be prevented by vaccination. The target of these vaccines is high-risk HPV genotypes that account for the majority of the cases of cervical cancers worldwide. The burden of cervical cancer is reduced by the administration of these vaccines which target HPV16 and 18 combined in a bivalent called Cervarix and a quadrivalent vaccine, which also includes HPV6 and 11, called Gardasil [21]. The Nigerian Federal Ministry of Health (FMH) has recommended that those between the ages of 9 and 26 years should receive HPV vaccines before the commencement of any sexual activity; however, these vaccines are not currently provided during free routine immunization programs [22]. They are available through purchase for individual use [23]. In order to draw attention to the need for concerted HPV vaccination efforts, this study aimed to determine the prevalence of genital HPV infection with its associated risk factors among women in Lagos, Nigeria.

## 2. Materials and Methods

### 2.1. Study Area and Design

This is a hospital-based cross-sectional study carried out among women who visited the gynecology clinic of the Lagos University Teaching Hospital (LUTH), Idi-araba, Lagos between July 2017 and June 2018. LUTH is a foremost federal-government-owned tertiary health institution in Lagos that offers specialty and subspecialty care including gynecological oncology services such as Papanicolaou (Pap) tests, human papillomavirus (HPV) testing, and colposcopy for cervical cancer screenings. The hospital acts mainly as a referral center for other government-owned and private hospitals in Lagos State. Lagos State is the commercial hub of Nigeria which has a population of almost 20 million inhabitants.

### 2.2. Study Population

A total of 180 participants were recruited into the study out of which only 15 participants did not give their written informed consent. A total of 165 consecutively consenting, sexually active women aged 18 years and above with or without cervical cytological abnormalities were enrolled in the study during their routine cervical cancer screening (Pap smear) at the gynecology and cytology clinics of the hospital. Women with ongoing pregnancy or within 6 months of childbirth, those who had undergone treatment for benign or malignant lesions of the cervix, those who were physically or mentally unable to give consent or undergo an interview, and those with a current cervical cancer diagnosis or on anticancer therapy were excluded from participation in the study. Interviewers used questionnaires to collect data on sociodemographic, sexual, and clinical data with confirmation of details from participants’ clinic records. All participants’ data were anonymized throughout the study.

### 2.3. Specimen Collection and Laboratory Analysis

A cervical brush was used to collect samples of exfoliated cervical cells from the cervical os of all the study participants after inserting a Cusco’s speculum into the vagina to expose the cervix. The cervical brush was then inserted into a liquid-based cytology medium (Regneix Labs, MurandMur, India) and vortexed to dislodge the cells into the medium. Exfoliated cervical cells were prepared from the liquid-based medium according to the kit manufacturer’s guide for the Papanicolaou test (Regenix Labs, MurandMur, India). The smears were made at two points on prelabelled glass slides and allowed to dry. The slides were delivered to the cytopathology laboratory in the Department of Morbid Anatomy and Histopathology, Lagos University Teaching Hospital for staining and microscopic examination. A 2 mL aliquot of the cervical cells in the liquid-based medium was transported on ice to the virology laboratory of the Central Research Laboratories, University of Ilorin and stored at −20 °C until DNA extraction and HPV studies were carried out.

Genomic DNA was extracted from each of the samples collected using a commercially available DNA extraction kit (Thermo Scientific, Waltham, MA, USA) according to the manufacturer’s instructions. The consensus region of HPV DNA was amplified by nested PCR using primers targeting the L1 gene region (one forward primer (MY09) and reverse primer (MY11) for the first amplification and one forward primer (GP5+) and reverse primer (GP6+) for the nested amplification). The cycling conditions for nPCR were preceded by an initial denaturation of 95 °C for 9 min, followed by 40 amplification cycles of 95 °C for 30 s, while annealing was for 2 min each at 50 °C and 45 °C, respectively, for the first and nested round of amplifications with an extension at 72 °C for 1 min 30 s and a final extension at 72 °C for 4 min. The size of the PCR products that were generated with MY09/11 and GP5+/6+ were 450bp and 150bp, respectively, as described by Hammou et al. [24]. The amplified DNA products were run on 1.5% agarose gel during electrophoresis and visualized using InGenius 3 ™ +System (Syngene, Frederick, MD, USA).

### 2.4. Data Management and Analysis

Data were double entered into Microsoft Excel 2016 (Microsoft Corporation, Redmond, WA, USA) for cleaning and imported into IBM SPSS statistic version 21 software (IBM Corp, Armonk, NY, USA) for analysis. Data analyses were performed using IBM SPSS statistic version 21 software for Windows. The chi-square test was used to determine differences between young (≤30 years) and middle-aged women (>30 years), and a one-sided probability of <0.05 was considered statistically significant. Bivariate logistic regression was used to identify factors associated with HPV positivity, with *p* values of <0.05 considered as statistically significant. Multivariate logistic regression analysis was not used because there were not enough variables that were significant in the bivariate analysis.

### 2.5. Ethical Considerations

The approval to conduct this study was obtained from the Health Research Ethics Committee of the Lagos University Teaching Hospital (ADM/DCST/HREC/APP/1603). Informed consent was obtained from each potential participant before inclusion in this study. The ethical principles of the Declaration of Helsinki were observed throughout the study.

## 3. Results

### 3.1. Participants’ Sociodemographic Characteristics

The age of study participants ranged from 18 to 65 years with a mean age of 41.38 ± 8.1 years. The median age of participants was 40 (interquartile range, 34–47) years. The majority of participants were within the age group of 25–54 years (83.0%), which consists more of married women (76.4%) and in a monogamous marriage (94.4%). There was greater representation among those who had tertiary education (77%) while only 2.4% had no formal education. Alcohol use was recorded among 19.4% of the participants.

The age of participants at first sexual experience ranged from 11 to 49 years with a mean age of 20.4 ± 3.3 years. It was observed that the majority of the women had their sexual debut at or below 16 years of age (38.8%) compared to those who had sexual initiation at ≥21 years (30.9%). There were more participants who had between two and four sexual contacts than those who had five or more sexual contacts.

### 3.2. Prevalence of HPV

Of the 165 cervical samples of participants screened for HPV in the study population, the prevalence of HPV was 81.82% (135/165) in all women and 87.59% (113/129) in women with normal cytology. The prevalence of HPV among women ≤ 30 years was 90.47% (19/21), while the prevalence among women > 30 years was 80.56% (116/144) (χ^2^ = 1.213; *p*-value = 0.271). Forty samples were HPV DNA positive (24.2%) using the MY09/11 primers, while one hundred and twenty-six samples (76.4%) were positive for HPV DNA when GP5+/6+ primers were used in a nested PCR (nPCR). A total of thirty-two samples (19.4%) were positive for both MY09/11 and GP5+/6+ consensus primers with an amplicon size of 450bp and 150bp, respectively.

**Figure 1:**
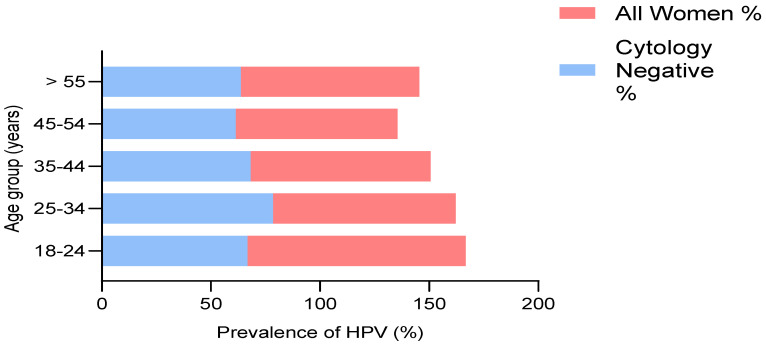
Age-specific detection of HPV DNA among all participants and cytology-negative women. The highest prevalence among all women was reported in the age group of 18–24 years, while the age group of 45–54 years had the lowest prevalence. HPV prevalence was high among cytology-negative women between the ages of 25 and 34 compared to the age group of 45–54 which had the lowest prevalence of all groups.

The age-specific HPV prevalence rates for women with normal cytology and all women in this study are shown in Figure 1. HPV prevalence was high among those within the age group of 18–24 years, although the number of participants was six (100%). The prevalence of HPV was more than 82% among the age groups of 25–34 and 35–44 years (31/37; 57/69), whereas in the age group of 45–54 years the HPV prevalence was approximately 74% (23/31).

The relationship between the sociodemographic variables for all participants and those with negative Papanicolaou (Pap) results is depicted in Table 1. There was a greater than 1% likelihood of contracting HPV infection among persons aged between 25 and 44 years, while above the age of 44 years there was a decline in the risk of infection among the study population. Those that were separated and those not yet married were at greater risk of HPV infection in this study. Most of the women that had primary and secondary education had a greater risk of HPV infection when compared with those who had tertiary education. However, it was observed that alcohol use contributed positively to the risk of HPV infection only among participants who had negative Pap smears in this study.

Table 2 shows the relationship between HPV positivity and sex-behavior-related variables. There was an observed association among those who had their first sexual experience at age 21 and above. Infection risk increased among those who had their first pregnancy in the age groups of 17–19 years and 20–24 years. There was a decline in the risk of HPV infection as the number of pregnancies increased among those who had normal cytology and all the women in this study. On the other hand, there was an increased risk of HPV infection when the number of lifetime sexual partners increased above four. There was a significant reduction of 40% in infection risk when condoms were used in the study population.

The relationship between HPV positivity and clinical characteristics variables is shown in Table 3. Those who had a history of sexually transmitted infections, those who had a history of genital warts, and those who used hormonal contraceptives had a greater likelihood of HPV infection in the study population. No association was observed between the history of cancer in the family and HPV infection.

## 4. Discussion

This study reported the prevalence of HPV in Lagos to be 81.82% among all participants in the study population and 87.59% among women without abnormal lesions in their cervix. This study has one of the highest prevalence rates reported from any part of Nigeria. This implies that less than 20% of sexually active women in Lagos, Nigeria have not been exposed to the virus already; therefore, the implementation of control measures and vaccination to mitigate the continuous spread of HPV among the younger women should be prioritized. The prevalence of HPV among young women (≤30 years) was higher when compared with middle-aged women (>30 years) in this study, although the difference was not statistically significant (χ^2^ =1.213; *p*-value = 0.271). Reports exist on the prevalence of HPV in Nigeria among different populations. In Lagos, HPV prevalence rates of 30.4%, 36.5%, and 37.5% have been reported, which is lower than the prevalence obtained in this study [17,18,25]. Other studies in Nigeria have reported a prevalence of 14.7% in Irun [15]; 26.3% in Ibadan [14]; 37% in Abuja [26]; 48.1% in Gombe [27]; and 76% in Kano [28].

Reports from a meta-analysis of HPV among Nigerian women that reviewed 17 studies in six geopolitical zones of the country reported a prevalence of 29% among studies that detected HPV [29]. According to regional stratification, Anoruo, Bristow, Mody, and Klausner [29] reported the highest seroprevalence of 71% in the southeast zone and produced the different prevalence reports with differing methods of detection. The result obtained from this study is higher than the prevalence obtained in the meta-analysis when HPV detection is considered, and the variations in the methods used in sample collection as well as HPV detection may account for this difference. However, the highest seroprevalence obtained in the southeast zone gives an indication of the prevalence determined in this study.

The prevalence obtained in this study was higher than in other communities in sub-Saharan Africa where similar population groups were considered. In rural Ethiopia, Leyh-Bannurah et al. [30] reported a prevalence of 17.3%. The study by Blossom et al. [31] in Kampala, Uganda reported the prevalence of HPV to be 46.2%. In a study conducted in South Africa, the prevalence of HPV was 74.0% among HIV-positive and 36.7% among HIV-negative women [32]. A study in Harare, Zimbabwe reported HPV prevalence to be 72% among women who were screened for cervical cancer [33].

The disparity in this study when compared with other studies may be because this study was conducted among women who were present for cervical cancer screening at a tertiary health facility in Lagos, and it is presumed that many of the patients were referred by healthcare workers following complaints of symptoms suggestive of either genital tract infection or cervical cancer. Moreover, the result obtained in other studies may be due to the sensitivity of the HPV assay used; existing variations in the different study populations due to varying risk exposures based on diverse sociocultural and geographical differences; and coinfection with HIV.

The HPV prevalence figure of 97.2% among HIV-infected individuals reported by Sahasrabuddhe, Mwanahamuntu, Vermund, Huh, Lyon, Stringer, and Parham [13] in Zambia, which is higher than the prevalence obtained in this study, seems to be one of the highest prevalence rates reported in sub-Saharan Africa. The dynamics of HPV transmission may reflect high-risk sexual practices among women in the Zambian study. Furthermore, it is thought that the high prevalence among HIV-infected individuals may reflect the failure of the immune system to clear HPV which may lead to chronic HPV infection. Although this study did not screen for HIV among the women recruited, it is suggested that HIV may play a role in the prevalence of HPV among this population.

This study found the highest prevalence rate in the age group of 18–44 years, though there was a similarity in HPV prevalence across all age groups. There was a decline in the likelihood of HPV infection in the age group of 25–34 years when compared to women aged 35–44 years in this study. This may be related to the sexual transmission and initiation of sexual activity of women in this age group. However, a high prevalence of HPV was found among women above 55 years old. This may reflect cumulative lifetime exposure and immune system vulnerability to the persistence or reactivation of latent infection [34].

There was a relationship between marital status and HPV infection in this study. Women who were either separated or unmarried were more prone to HPV infection in this study. This is understandable because it has been postulated that these groups tend to date new partners which puts them at an increased risk of HPV infection. The study by Nejo et al. [35] reported the highest prevalence among divorced women, which is similar to this study. However, the majority of the women recruited into this study belong to the tertiary education class. There was a greater risk of HPV infection among women that had primary and secondary education in this study. It has been observed that this level of education has been associated with high-risk sexual behavior and a poor attitude to seeking medical attention in the event of acquiring a sexually transmitted infection [36].

In the current study, there was a higher risk of exposure to HPV among women who had their first sexual experience around the age of 21 years, which is an indication that the late onset of sexual activity does not limit the risk of HPV infection later in life. It is believed that most young women have their first sexual experience during adolescence, although that was not observed in this study. Moreover, based on findings in this study, there was an increased likelihood of HPV infection among those who had their first pregnancy between the ages of 17 and 24 years. Other studies have also reported that early sexual debut and childbirth at an early age could increase the likelihood of HPV infection. This present study found that only childbirth at an early age could be relied on as a representation of the onset of sexual activity among women in our study environment because it is conceivable that some women may have underreported the age of sexual debut [17,18,35].

There was an increased risk of HPV infection when there were more than four lifetime sexual partners. On the other hand, a significant reduction in HPV infection risk was observed when condoms were used by women in this study. This further emphasizes the importance of the use of condoms as a means of protection during sexual intercourse to prevent sexually transmitted infections such as HPV. This is similar to the findings of Veldhuijzen et al. [37] who reported that exposure to HPV could be linked to varying sexual behaviors such as early sexual debut, a high number of recent or current sexual partners, the behavior of the sexual partners, and a high number of lifetime sexual partners. However, this present study differs from the work of Thomas, Herrero, Omigbodun, Ojemakinde, Ajayi, Fawole, Oladepo, Smith, Arslan, and Munoz [14] who reported that condom use was unrelated to HPV prevalence in their study.

On consideration of some past clinical history, it was observed that those who had a history of STI or genital warts and those who had used hormonal contraceptives had a greater likelihood of infection with HPV. This result agrees with the findings of Clarke et al. [38] who reported a significant association between HPV infection and the use of birth control pills. It stands to reason that if an individual has a history of sexually transmitted infection or genital warts it indicates the possibility that she would have been exposed to HPV at some point in time. Ojiyi et al. [39] in Maiduguri reported that there were no significant associations between HPV and past sexually transmitted infections. The authors related their findings to the reluctance of most women to disclose details of previous sexually transmitted infections or their treatment. However, this was not the case in the urban population considered in our study. This present study did have some limitations: it was hospital-based, which could have contributed to the prevalence obtained, and we did not determine the HIV status of the women at recruitment.

## 5. Conclusions

The high prevalence of HPV reported in this study is a pointer to the fact that there is an increased transmission of HPV among sexually active women in Lagos, Nigeria. There were associations with risk factors, such as young age, marital status, alcohol use, a higher number of lifetime sexual partners, and a history of genital warts, identified in this study. Significant associations were observed in this study among women who had a history of STI, those who had their first pregnancy around the age of 25 years, and those who used condoms. Therefore, there is a need for educating the public about HPV testing, vaccination, and early detection tests for cancer prevention in Lagos, Nigeria.

## Figures and Tables

**Table 1:** Odds ratios of HPV positivity according to sociodemographic variables.

		All Participants			Cytology-Negative Participants		
Variables	Categories	N/P 30/135	OR (95% CI)	*p*-Value	N/P 16/113	OR (95% CI)	*p*-Value
Age (years)	18–24	0/6	-	-	0/4	-	-
	25–34	6/31	1.15 (0.29–4.62)	0.85	6/29	0.35 (0.04–3.15)	0.35
	35–44	12/57	1.06 (0.30–3.68)	0.93	6/47	0.56 (0.06–5.05)	0.60
	45–54	8/23	0.64 (0.17–2.46)	0.52	3/19	0.45 (0.04–4.82)	0.51
	≥55	4/18	1.0 (referent)		1/14	1.0	
MS	Married	23/103	1.0		13/86	1.0	
	Divorced/Widowed/Separated	3/15	1.05 (0.32–3.43)	0.93	0/14	1.53 (0.38–0.69)	0.55
	Not married	4/17	1.18 (0.23–6.13)	0.85	3/13	-	-
ES	No schooling	1/3	1.0		0/1	1.0	
	Primary	1/6	2.00 (0.09–44.35)	0.66	0/4	1.0	-
	Secondary	4/23	1.92 (0.16–23.35)	0.61	2/17	-	-
	Tertiary	24/103	1.43 (0.14–14.36)	0.76	14/91	-	-
Alcohol use	No	23/110	1.0		14/95	1.0	
	Yes	7/25	0.75 (0.29–1.93)	0.55	2/18	1.33 (0.28–6.34)	0.72
Smoking	No	30/133	1.0		16/111	1.0	
	Yes	0/2	-	-	0/2	-	-

N: negative; P: positive; MS: marital status; ES: educational status; - means nil.

**Table 2:** Odds ratios of HPV positivity according to sex-behavior-related variables.

		All Participants			Cytology-Negative Participants		
Variables	Categories	N/P 30/135	OR (95% CI)	*p*-Value	N/P 16/113	OR (95% CI)	*p*-Value
AFS (years)	≤16	12/48	1.0		5/43	1.0	
	17–20	10/30	0.75 (0.29–1.95)	0.55	9/22	0.28 (0.08–0.95)	0.04
	≥21	8/57	1.78 (0.67–4.72)	0.25	2/48	2.79 (0.51–15.13)	0.23
AFP (years)	<17 years	7/34	1.0		2/28	1.0	
	17–19 years	3/27	1.85 (0.44–7.85)	0.40	2/22	0.79 (0.10–6.03)	0.82
	20–24 years	1/17	3.50 (0.40–30.80)	0.26	0/13	-	-
	≥25 years	7/5	0.15 (0.04–0.60)	0.01	4/4	0.07 (0.01–0.52)	0.01
Parity	Not pregnant	5/22	1.0		4/20	1.0	
	1–4 pregnancies	13/68	1.19 (0.38–3.71)	0.77	6/60	2.00 (0.51–7.81)	0.32
	≥5 pregnancies	12/45	0.85 (0.27–2.72)	0.79	6/33	1.10 (0.28–4.38)	0.89
LSP	1	12/56	1.0		8/52	1.0	
	2–4	15/62	0.89 (0.38–2.05)	0.78	7/49	1.08 (0.36–3.19)	0.89
	≥5	3/17	1.21 (0.31–4.81)	0.78	1/12	1.85 (0.21–16.19)	0.58
Condom use	No	14/92	1.0		6/82	1.0	
	Yes	16/43	0.41 (0.18–0.91)	0.03	10/31	0.23 (0.08–0.68)	0.01

N: negative; P: positive; AFS: age at first sex; AFP: age at first pregnancy; LSP: lifetime sex partners; - means nil.

**Table 3:** Odds ratios of HPV positivity according to clinical characteristics variables.

		All Participants			Cytology-Negative Participants		
Variables	Categories	N/P 30/135	OR (95% CI)	*p*-Value	N/P 16/113	OR (95% CI)	*p*-Value
Clinical Characteristics							
HSTI	No	24/68	1		14/54	1	
	Yes	6/67	3.94 (1.51–10.25)	0.005	2/59	7.65 (1.66–35.21)	0.01
HGW	Absent	27/112	1		16/95	1	
	Present	3/23	1.85 (0.52–6.61)	0.34	0/18	-	-
HCU	No	26/116	1		16/100	1	
	Yes	4/19	1.06 (0.33–3.39)	0.92	0/13	-	-
HCF	No	26/120	1		16/101	1	
	Yes	4/15	0.81 (0.25–2.65)	0.73	0/12	-	-

N: negative; P: positive; HSTI: history of sexually transmitted infection; HGW: history of genital warts; HCU: hormonal contraceptive use; HCF: history of cancer in the family; - means nil.

## Data Availability

The data presented in this study are available on request from the corresponding author.
